# Spatially-Resolved Top-down Proteomics Bridged to MALDI MS Imaging Reveals the Molecular Physiome of Brain Regions*[Fn FN2]

**DOI:** 10.1074/mcp.M116.065755

**Published:** 2017-11-09

**Authors:** Vivian Delcourt, Julien Franck, Jusal Quanico, Jean-Pascal Gimeno, Maxence Wisztorski, Antonella Raffo-Romero, Firas Kobeissy, Xavier Roucou, Michel Salzet, Isabelle Fournier

**Affiliations:** From the ‡Laboratoire Proteomique, Réponse Inflammatoire et Spectrométrie de Masse (PRISM) - INSERM U1192, Université Lille 1, Bât SN3, 1^er^ étage, Cité Scientifique, F-59655 Villeneuve d'Ascq Cedex, France;; §Département de Biochimie Lab. Z8–2001, Faculté de Médecine et des Sciences de la Santé, Université de Sherbrooke, Sherbrooke, Canada;; ¶Department of Biochemistry and Molecular Genetics, Faculty of Medicine, American University of Beirut, Beirut, Lebanon

## Abstract

Tissue spatially-resolved proteomics was performed on 3 brain regions, leading to the characterization of 123 reference proteins. Moreover, 8 alternative proteins from alternative open reading frames (AltORF) were identified. Some proteins display specific post-translational modification profiles or truncation linked to the brain regions and their functions. Systems biology analysis performed on the proteome identified in each region allowed to associate sub-networks with the functional physiology of each brain region. Back correlation of the proteins identified by spatially-resolved proteomics at a given tissue localization with the MALDI MS imaging data, was then performed. As an example, mapping of the distribution of the matrix metallopeptidase 3-cleaved C-terminal fragment of α-synuclein (aa 95–140) identified its specific distribution along the hippocampal dentate gyrus. Taken together, we established the molecular physiome of 3 rat brain regions through reference and hidden proteome characterization.

On-tissue spatially-resolved proteomics provides a direct means to examine proteomic fluctuations at the cellular level in response to changes in the tissue microenvironment (1). Its importance is evident in physiopathological diseases such as cancer, where proteomic analysis of the complete tissue does not take into account tumor heterogeneity and thus the cellular cross-talks occurring in different regions of the tumor (2–8). Combined with MALDI mass spectrometry imaging (MSI)^1^, which can map the distribution of molecules (9, 10), on-tissue spatially-resolved proteomics can provide details of the molecular events occurring at cellular level in such discrete regions. In this context, our team made an ongoing effort to develop microscale techniques that can achieve reliable identification by shot-gun proteomics and quantification of proteins within an area of the most limited size, and correlate these expression changes with alterations in cell phenotypes and/or biological state (1, 11, 12).

Liquid microjunction (LMJ) microextraction was the first technique developed for this purpose (11, 13–24). LMJ is the application of a droplet (1–2 μl) of solvent on top of a locally digested area, in order to extract peptides after on-tissue trypsin digestion. About 1500 protein groups from a tissue area of about 650 μm in diameter corresponding to less than 1900 cells can be identified (1). A method providing automatic microextraction and injection into the nanoLC-MS instrument from a tissue surface for shotgun microproteomics was also implemented. Thus an online LMJ coupling to on-tissue digestion using automatic microspotting of the digestion enzyme allows the analysis of a very limited area of the tissue section down to 250 μm spot size (corresponding to an equivalent average number of 300 cells) (25). Application to ovarian cancer resulted in the identification of 1148 protein groups (12).

Parafilm-Assisted Microdissection (PAM) consists of mounting the tissue on a glass slide covered with a stretched layer of Parafilm M™ (17, 26, 27). Regions of interest previously highlighted by MALDI-MSI are then manually microdissected. The microdissected areas are then submitted to in-solution digestion and nanoLC-MS/MS, allowing the identification and relative quantification of many proteins (17). Application to prostate cancer biomarker discovery led to the identification of 1251 proteins, 485 of which fit the Fisher's test criterion. 135 were upregulated and 73 downregulated in 8 prostate cancer biopsies (27).

All these strategies based on bottom-up proteomics remain limited as it is difficult to determine whether the protein is in its native or truncated form. Also, there is no direct information about post-translational modifications (PTMs), which often require specific enrichment steps. The top-down proteomics approach gives a unique solution for intact protein characterization with applications to monoclonal antibody characterization; *de novo* sequencing and PTM elucidation without any conventional PTM-specific enrichment usually applied for bottom-up strategies and has already proven disease-monitoring capabilities for various pathologies (28–33). However, this approach usually needs large amounts of protein samples and extensive fractionation techniques to be competitive with conventional bottom-up strategies in terms of unique protein IDs, mostly because of the need for accumulation of more microscans required for intact protein MS and MS/MS to generate spectra suitable for analysis. The molecular weight distribution tends to be restricted to lower molecular weight products as it remains challenging for the mass analyzer to measure the exact mass of high molecular weight compounds. Currently, top-down proteomics gives great opportunities for the better understanding of biological mechanisms and has been used complementary to bottom-up proteomics to gain information about PTMs, intact molecular weight and truncated forms of proteins, all of which can be critical for biomarker hunting. However, its association with tissue MALDI-MSI and clinical investigations remains rare but promising (34, 35). Notably, one study involving on-tissue extraction and direct infusion of protein extracts permitted the detection of a specific proteoform in nonalcoholic steatohepatitis patient tissues that could not be reliably identified by the bottom-up approach, showing great promises for disease characterization (34, 35).

Recently, it has been shown that the proteome of higher mammals might has been under evaluated. We recently demonstrated the presence of several proteins issued from a mature mRNA that is normally assumed to contain a single coding DNA sequence (CDS). These proteins, so-called alternative proteins (also known as microproteins, micropeptides and SEPs), are issued from alternative open reading frames (altORFs) (also known as smORFs and sORFs) and correspond to the hidden proteome (36). AltORFs are defined as potential protein-coding ORFs exterior to, or in different reading frames from, annotated CDSs in mRNAs and ncRNAs. Indeed, proteins translated from nonannotated altORFs were detected in several studies by MS (36, 37). AltORFs are present in untranslated mRNA regions (UTRs) or overlap canonical or reference ORFs (refORFs) in a different reading frame. Thus, alternative proteins are not identical to reference proteins (36, 37). For example, AltMRVI1, an alternative protein of the *MRVI1* gene present in the 3′UTR region of the MRVI1 mRNA, has been shown to interact with BRCA1 (36). Translation of altORFs in human mRNAs in addition to refORFs provides access to a large set of novel proteins whose functions have not been characterized, and that cannot be detected using conventional protein databases. Moreover, conventional bottom-up proteomics is not well suited for their analysis because these proteins are relatively small (between 2 and 20 kDa) and more often do not contain enzyme-cleavable sites. Thus, the number of enzymatically cleaved peptides generated is too small compared with those of reference proteins. Consequently, the probability of peptide and protein identification is poor, in the absence of low-mass protein enrichment steps. In this context, top-down proteomics offers better capabilities to detect alternative proteins, considering that no enzymatic digestion steps are used and this strategy is well suited to low-mass proteins.

In this article, further investigation of the hidden proteome on biological tissues was done. For this purpose, we developed a novel strategy based on MALDI-MSI coupled to on-tissue spatially-resolved top-down proteomics to identify low-mass proteins and to localize them. We performed our analyses on rat brain to compare the reference proteome and the hidden proteome in different regions. Differential distributions of unique and common biological and functional pathways among the three different regions were then determined. A direct link can be drawn between the classes of proteins identified and the biological functions associated with each specific brain region. Interestingly, we identified different large peptide fragments from either neuropeptide precursors or from constitutive synapse proteins. These large peptides are different in each brain region and are in line with the presence of specific endocrine processing enzymes like prohormone convertases (38), neutral endopeptidases (39), or angiotensin converting enzymes (40, 41).

We also showed the presence of specific PTMs associated to each brain region and in relation with their local function. Moreover, we demonstrated the presence of novel proteins issued from alternative ORFs and specific for each brain region. Finally, we performed back correlation between the identified proteins and their relative quantification at a given cellular localization with MALDI-MSI. Taken together, we could depict a molecular proteomic pattern in three different rat brain regions in relation with the biological and physiological functions of each specific brain area.

## EXPERIMENTAL PROCEDURES

### 

#### 

##### Experimental Design and Statistical Rationale

We first acquired MS images of lipids. These images were subjected to spatial segmentation to identify regions of interest (ROIs) that can be subjected to LMJ or PAM spatially-resolved proteomics. For this purpose, several tissue sections were obtained from rat brain. LMJ and PAM were followed by top-down proteomics for protein identification from 3 different brain regions. Back correlation by MALDI-MSI was then performed (*n* = 3). Reference and alternative proteins were thus identified and localized in the 3 rat brain regions.

##### Chemicals

MS grade water (H_2_O), acetonitrile (ACN), methanol (MeOH), ethanol (EtOH) and chloroform were purchased from Biosolve (Dieuze, France). The cleavable detergent ProteaseMAX was purchased from Promega (Charbonnieres, France). Parafilm M, 2,5-dihydroxybenzoic acid (DHB), sinapinic acid (SA), α-cyano-4-hydroxycinnamic acid (HCCA), aniline, sodium dodecyl sulfate (SDS), dl-dithiothreitol (DTT), trifluoroacetic acid (TFA) and formic acid (FA) were purchased from Sigma (Saint-Quentin Fallavier, France).

##### Tissues

Male Wistar rats of adult age were sacrificed by CO_2_ asphyxiation and dissected. Brain tissues were frozen in isopentane at −50 °C and stored at −80 °C until use.

##### Tissue Section Preparation

For MALDI-MSI experiments, tissues were cut in 10 μm slices using a cryostat (Leica Microsystems, Nanterre, France) and were mounted on Indium Tin Oxide (ITO) coated glass slides (LaserBio Labs, Sophia-Antipolis, France) by finger-thawing. For LMJ and PAM, MSI-adjacent tissue slices were cut at 30 μm thickness. For LMJ, the tissues were mounted on polylysine glass slides (Thermo Fisher Scientific, Courtaboeuf, France) whereas for PAM, the tissues were mounted on Parafilm M-covered polylysine glass slides (17). After tissue section preparation, the slides were immediately dehydrated under vacuum at room temperature for 20 min. The slides were then scanned and stored at - 80 °C until use.

##### Intact Protein Extraction Buffer

To ensure little-to-no protein hydrolysis by endogenous proteases, every step from buffer preparation to nanoLC-MS/MS analysis were carried out within the same day with on-ice conservation in between sample processing steps. A 1% solution of temperature- and acid-cleavable commercial detergent (ProteaseMAX) was prepared in 50 μm DTT and was aliquoted and immediately stored at −20 °C until use according to manufacturer's recommendations. The aliquots were processed the same day of sample extraction to ensure minimal degradation of the detergent over time. An aliquot was further diluted in ice-cold 50 μm DTT to obtain a final detergent concentration of 0.1% and stored on ice until use. Each aliquot was used within the day without conservation of the remaining solution.

##### LMJ Experiments

To ensure optimal protein extraction, lipids were depleted from the tissue section by immersing the glass slides in consecutive solvent baths consisting of 70 and 95% EtOH (1 min each time) and chloroform (30 s) with complete solvent evaporation under reduced pressure at room temperature between each washing step. The slides were then re-scanned to obtain better optical images with better contrast as the washing steps improve the visibility of the structures on the tissue section. The tissue slide for LMJ extraction was placed on a TriVersa NanoMate instrument (Advion, Ithaca, NY). Proteins were then extracted from every ROI by completing six cycles of extraction consisting of pipetting up 1.5 μl of detergent solution, dispensing 0.8 μl of extraction buffer on the surface of the selected ROI with 10 iterations of up-and-down pipetting, aspiration of 2.5 μl by the device and expulsion of 4 μl from the pipette tip into a clean tube to ensure complete retrieval of the initial 1.5 μl volume for each cycle. Per ROI, the final collected volume was 9 μl; the extracts were immediately placed on ice until further processing.

##### PAM Experiments

10 μl of extraction buffer was transferred into a tube. Selected ROIs were manually dissected using a clean scalpel blade and transferred into the protein extraction buffer. Excision of the ROIs was performed with the aid of a microscope. The samples were placed on ice until further processing.

##### nanoLC-MS/MS

The extracts obtained using either the LMJ or PAM approaches were sonicated for 5 min and incubated at 55 °C for 15 min to ensure reduction of disulfide bonds. These were then quickly centrifuged to rally condensation droplets at the bottom of the tube. The parafilm pieces were then carefully removed from the tubes using a pipette tip and the tubes were then heated at 95 °C for 10 min to ensure complete detergent dissociation. The tubes were then quickly centrifuged and placed on ice. 11 μl of 10% ACN in 0.4% FA in water were added to each tube to obtain a final ACN concentration like initial LC gradient conditions and the samples were stored at 4 °C until nanoLC-MS/MS analysis on the same day.

5 μl of each sample was loaded onto a 2 cm X 150 μm internal diameter (i.d.) PLRP-S (Varian, Palo Alto, CA) IntegraFrit sample trap-column (New Objective, Woburn, MA) at a maximum pressure of 280 bar using a Proxeon EASY nLC-II chromatographic system (Proxeon, Thermo Scientific, Bremen, Germany). Proteins were separated on a 15 cm X 100 μm diameter i.d. PLRP-S column with a linear gradient of ACN from 5 to 100% and a flow rate of 300 nL/min. 10 μl of the samples were also injected and separated using a 3-h gradient.

Data were acquired on a Q-Exactive mass spectrometer (Thermo Fisher Scientific, Bremen, Germany) equipped with a nanoESI source (Proxeon, Thermo Fisher Scientific, Bremen, Germany). 1.6 kV was applied on the PicoTip nanospray emitter (New Objective) and the spectra were acquired in data-dependent mode using a top 3 strategy. Full scans were acquired by averaging 4 microscans at 70,000 resolution (at *m*/*z* 400) within a *m/z* range of 800–2000 with an AGC target of 1 × 10 ^6^ and a maximum accumulation time of 200 ms. The three most abundant ions with charge states superior than +3 or unassigned were selected for fragmentation. Precursors were selected within an m/z selection window of 15 by the quadrupole and fragmented by averaging two microscans at a resolution of 70,000 with a Normalized Collision Energy (NCE) of 25. The AGC target was set to 1 × 10 ^6^ with a maximum accumulation time of 500 ms. Dynamic exclusion was set to 20 s.

##### Data Analysis

RAW files were processed with ProSight PC 3.0 or 4.0 (Thermo Fisher Scientifique, Bremen Germany). Spectral data were deisotoped using the cRAWler algorithm and searched against the complex *Rattus norvegicus* ProSightPC database version 2014_07. Using a similar approach, a second search was performed to detect alternative protein products, by interrogating RAW files with a concatenated custom database containing every reference proteins and their isoforms. These were generated from an *in-silico* transcriptome-wide translated database that contains every possible reference and alternative protein products from the Ensembl Rnor 6.0 transcripts sequence database with at least 30 amino acids (36). For alternative protein identification, it was verified that the ID was coming from a specific precursor that was not identified during the reference protein search. Files were searched using a two-step search tree containing a 1-kDa precursor tolerant search (“Absolute”) and a “Biomarker” search and MS/MS spectra were matched with sequences within a 15-ppm mass tolerance. Proteins were considered identified when one of the two steps gave expected values (E-value) inferior to 1 × 10^−4^.

Likewise, data from PMID 27512083 (42) were interrogated using the same search strategy with the concatenated database to identify alternative proteins that were not interrogated in the original publication.

As ProsightPC's “Absolute” search mode adds multiple identifications for a single spectrum, output files were filtered using a custom R script. For each identified spectrum, 1) the one with the best E-Value and (2) identification that had the closest experimental mass compared with ProsightPC database was selected, which were concatenated in a single table. In this table, the ProsightPC PTMs were considered true if this PTM matches both its theoretical and experimental masses. On the other hand, mass shifts that matched known shifts were annotated accordingly (*e.g.* +80 for phosphorylation, +42 for acetylation) whereas undescribed shifts were automatically marked as unmodified (supplemental Data S1). Finally, a nonredundant identification file was generated (supplemental Data S2) containing information about identifications, methods, ROIs, found modifications, E-values, best P-score, and spectral-count.

The mass spectrometry proteomics data have been deposited to the ProteomeXchange Consortium via the PRIDE (43) partner repository with the data set identifier PXD005424.

##### Subnetwork Enrichment Pathway Analyses and Statistical Testing

Elsevier's Pathway Studio version 10.0 (Ariadne Genomics/Elsevier) was used to deduce relationships among differentially expressed proteomics protein candidates using the Ariadne ResNet database (44, 45). “Subnetwork Enrichment Analysis” (SNEA) algorithm was selected to extract statistically significant altered biological and functional pathways pertaining to each identified set of protein hits among the different groups. SNEA utilizes Fisher's statistical test set to determine if there are nonrandom associations between two categorical variables organized by specific relationships. Integrated Venn diagram analysis was performed using “the InteractiVenn”: a web-based tool for the analysis of complex data sets (46). See supplemental Data S3 and S4 for the listed differential pathways.

##### MALDI-MSI

DHB matrix (50 mg/ml in 6:4 v/v MeOH/0.1% TFA in water) was manually sprayed using a syringe pump connected to an electrospray nebulizer at a flow rate of 300 μl/h under nitrogen gas flow. The nebulizer was moved uniformly across the entire tissue until crystallization was sufficient to ensure optimal lipid detection. The tissue was then analyzed using an UltraFlex II MALDI-TOF/TOF mass spectrometer equipped with a Smartbeam Nd-YAG 355 nm laser and controlled by FlexControl software (Bruker Daltonics, Bremen, Germany). Acquisition was performed in positive reflector mode with an *m*/*z* range of 50 to 900 and a spatial resolution of 300 μm. Each image pixel was obtained by averaging 300 laser shots at a rate of 200 Hz. External calibration was performed using the Peptide calibration standard mix 6 (LaserBio Labs). Lipid ion distributions were generated using FlexImaging software version 3.0 (Bruker Daltonics).

For intact protein imaging, SA and HCCA liquid ionic matrices were used. These were prepared by dissolving the matrices in 7:3 v/v ACN/0.1% TFA in water containing 7.2 μl of aniline at a concentration of 15 and 10 mg/ml, respectively. The matrices were deposited on the tissue sections using ImagePrep (Bruker Daltonics). Images were acquired using the UltraFlex II instrument in positive linear mode with an *m*/*z* range of 3000–25000 and 2000–25000, respectively, at 50 μm resolution with the laser size set using “Medium” setting. Each image pixel was obtained by accumulating 500 laser shots at a rate of 200 Hz. External calibration was performed using the Protein Calibration standard I (Bruker Daltonics).

Image files were processed using SCiLS Software (version 2015b, SCiLS GmbH, Bremen, Germany). Baseline removal was performed by applying the tophat filter, and normalization was done based on total ion count (TIC). Peak detection was performed by orthogonal matching pursuit, and the peaks were aligned to the mean spectrum by centroid matching. The *m*/*z* intervals were set to ± 5 Da. Spatial segmentation was made using the bisecting k-means algorithm using Manhattan distance calculation. After analysis, the ROIs were determined by selecting regions where the correlation distances were significantly distant from one another. The ion images of the individual peaks were plotted following medium denoising and automatic hotspot removal.

For back-correlation between protein MALDI-MS and top-down proteomics identification, spectra underwent realignment after *m*/*z* intervals were defined at ± 5Da for both HCCA and SA images using SCiLS. The maxima of the *m*/*z* intervals obtained after peak detection (Observed M_avg_) were individually matched with the average masses (M_avgs_) of top-down-identified proteins derived from their measured monoisotopic masses (M_mono._). Matching was performed with ΔM_avgs_ ≤ 6 Da all throughout the measured mass range and by considering that MALDI MS mass deviations tends to increase with high molecular weight. When available, tissue brain *in situ* hybridization images from Allen Brain Atlas (47) were added to analysis (supplemental Data S7).

##### Tissue Immunofluorescence

Immunofluorescence was performed on 10-μm sagittal rat brain sections (supplemental Data S9). The sections were immersed in blocking buffer (PBS 1× containing 1% bovine serum albumin, 1% ovalbumin, 2% Triton, 1% NDS, and 0.1 m Glycine) for 1 h. The primary antibodies monoclonal mouse Anti-GFAP (1:500, Millipore, Molsheim, France), Anti-Stathmin (1:100, Abcam, Cambridge, UK), Anti-α-synuclein C-terminal (20 μg/ml, Abcam) and Anti-BASP1 (1:100, Abcam) were diluted with the blocking buffer and applied to the sections except for the negative control where only the blocking buffer was applied. The sections were then incubated overnight at 4 °C. The following day, the sections were washed three times with PBS 1x, and incubated for 1h at 37 °C with the secondary antibody Alexa fluor donkey anti-mouse (1:1000, Life Technologies, ThermoFisher Scientific, Courtaboeuf, France) for Anti-GFAP and Alexa fluor rabbit anti-mouse (1:2000, Life Technologies) diluted in blocking buffer without 0.1 m glycine. Afterward, the sections were further washed with several changes of PBS 1x, stained with Sudan black 0.3% for 10 min to decrease the background generated by lipids, and were eventually counterstained with Hoechst solution (1: 10,000). The slides were then washed with PBS 1×, and Dako fluorescent mounting medium was applied on the sections before putting cover slips. Confocal images were obtained using a confocal microscope (Leica Biosystems, Nussloch, Germany). Processing of the images was performed using Zen version and applied on the entire images as well as on controls.

## RESULTS

### 

#### 

##### Spatially-Resolved Top-Down Proteomics and MALDI-MSI

**Fig. 1.**
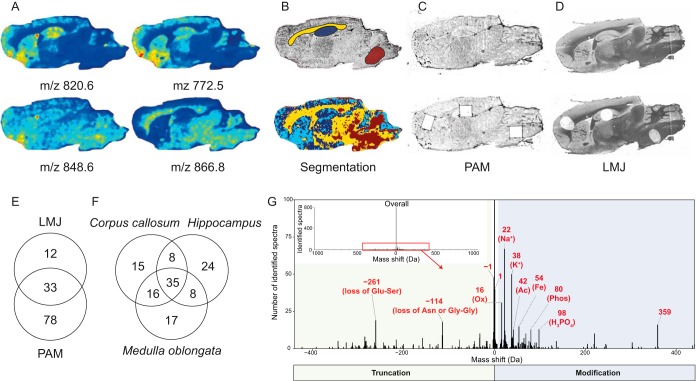
***A,* Molecular images after median normalization of spectra followed by medium denoizing and automatic hotspot removal.** *B*, Optical image with highlighted regions of interest *corpus callosum* (yellow), *hippocampus* (blue) and *medulla oblongata* (brown) (top) and spatial segmentation analysis using the Bisecting k-Means approach using Correlation as the distance metric (bottom). *C–D*, Optical images of PAM and LMJ tissue sections with the top and bottom panels showing the tissue sections before and after ROI processing, respectively. *E*, Venn Diagram of the extracted proteins per technique (LMJ or PAM) and (*F*) global unique identifications using both strategies. *G*, Overall mass shifts of observed proteins precursors *versus* their theoretical masses (*G*, inset) and most abundant observed mass shifts within a ± 400 Da tolerance window (*G*) with annotation of known mass shifts. −114 Da corresponds to loss of “Asn” at N-term of ATP synthase-coupling factor 6, mitochondrial or loss of “Gly-Gly” at C-term of Ubiquitin monomer and −261 corresponds to loss of Glu-Ser at C-term of Thymosin beta-4.

Different types of molecules can be used in MALDI-MSI to determine ROIs from biological tissues such as lipids, endogenous or tryptic peptides and proteins. However, lipid MALDI-MSI is the most convenient to our approach as it gives good spatial resolution and does not need extensive sample preparation steps. Our first developments were performed on rat brain tissue sections (Fig. 1). Different ROIs can be retrieved after lipid MALDI-MSI (Fig. 1*A*) followed by nonsupervised spatial segmentation analysis (Fig. 1*B*, bottom) compared with the optical image (Fig. 1*B*, top). Three ROIs in the *hippocampus*, *corpus callosum,* and *medulla oblongata* (Bregma Index lateral 1.90 mm) were selected for further processing as their segmentation profiles were sufficiently distinct.

Based on these selected ROIs, the two main strategies to perform spatially-resolved proteomics studies were then realized *i.e.* PAM (Fig. 1*C*) or LMJ (Fig. 1*D*). Based on the identified proteins, our approach mostly enables identification of low molecular weight (from 1.6 to 21.9 kDa) and most abundant proteins. These two strategies allowed the identification of proteins that were common within the three regions as well as specific ones. Analyses of the three ROIs gave a total of 123 proteins identified (Fig. 1*E* and 1*F*, supplemental Data S1 and S2). One hundred eleven proteins have been identified in PAM and 45 in LMJ. The number of specific proteins identified is higher with PAM than with LMJ, which might be related to tissue washing steps prior to protein extraction and smaller area of extraction. By combining the two approaches, 15 specific nonredundant proteins were identified from the corpus callosum, 17 from medulla oblongata, and 24 from hippocampus (Fig. 1*E* and 1*F*, supplemental Data S1 and S2). Thirty-five are common to the 3 brain regions; 16 are shared between *corpus callosum* and *medulla oblongata*, 8 between *corpus callosum* and *hippocampus*, and 8 between *medulla oblongata* and *hippocampus*. Most identified spectra exhibited a mass shift close to 0 Da (Fig. 1*G*, inset). The mass tolerant identification approach allowed characterization of modified forms of proteins, which can either be truncated compared with database prediction or modified (Fig. 1*G*) in a similar fashion to what is described by Chick *et al.* (48).

##### Systems Biology Analyses of the Identified Proteins

**Fig. 2.**
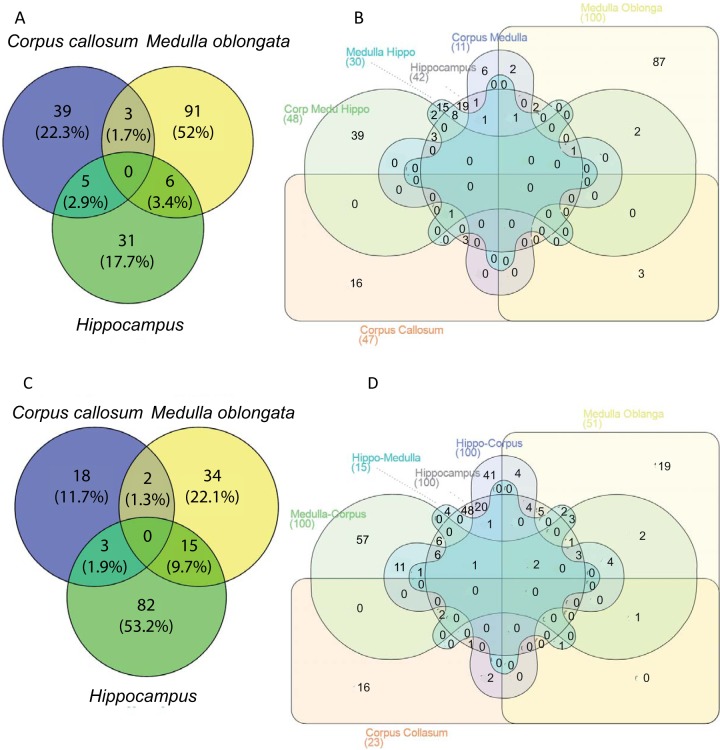
**Differential distribution of unique and common/intersected biological and functional pathways among the three brain regions (corpus callosum, hippocampus and medulla oblongata) obtained with LMJ (*A*) or PAM (*C*) extraction methods.** Each brain region was analyzed across the three regions using a comprehensive Venn analysis representation extracted from Subnetwork Enrichment Analysis (*B* with LMJ and D with PAM).

**Fig. 3.**
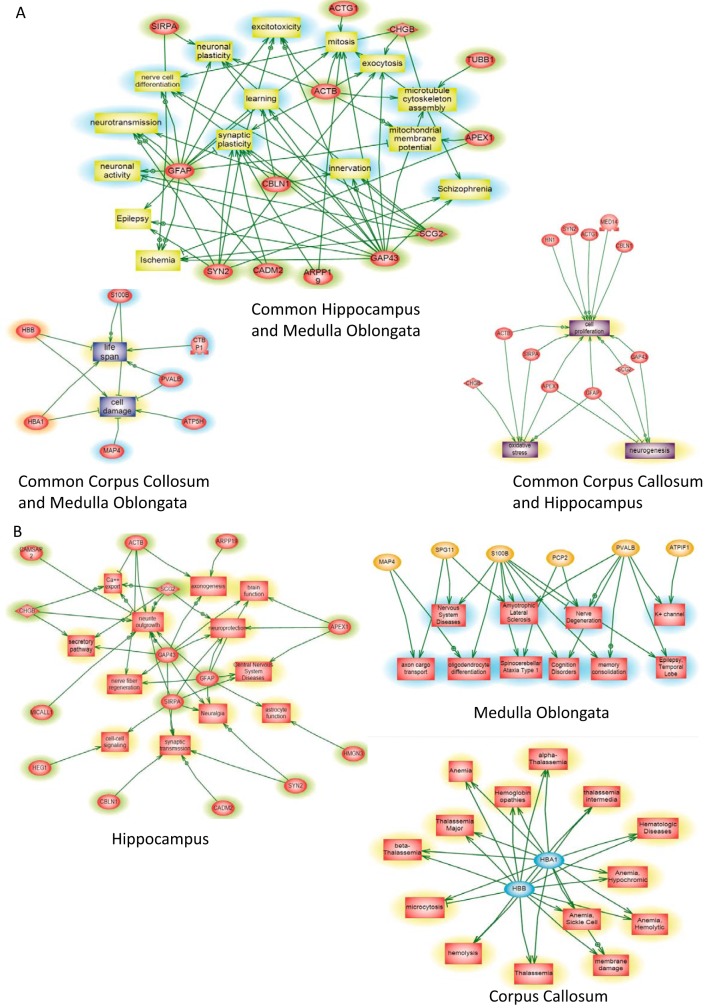
***A*, Global pathway analyses of the over-expressed proteins common to two different regions *i. e*.** *hippocampus* and *medulla oblongata*, *hippocampus* and *corpus callosum* and *medulla oblongata* and corpus callosum. *B*, Over-expressed proteins in the *hippocampus*, *medulla oblongata* or *corpus callosum* were involved in globally altered molecular pathways.

Functional enrichment analysis using Search Tool for Recurring Instances of Neighboring Genes (STRING, (49)) identified 4 GO terms associated with Molecular function: Hydrogen ions transmembrane transport (GO 0015078), Cytochrome-c oxidase activity (GO: 0004129), Ion transmembrane transporter activity (GO: 0015075), and Oxidoreductase activity (GO: 0016491). Systems biology analysis was then performed on the over-expressed proteins of each group for LMJ (Fig. 2*A*) and for PAM (Fig. 2C). Differential distributions of unique and common statistically significant biological and functional pathways among the three different regions are depicted in Fig. 2*A* for LMJ and 2*C* for PAM, including 39 *versus* 18 pathways for *corpus callosum*, 91 *versus* 34 pathways for *medulla oblongata* and 31 *versus* 82 pathways for *hippocampus* (Please refer to supplemental Data S3 for the identity of each of the unique pathways). Combined differential pathways were analyzed across the three regions. Three pathways in LMJ *versus* 2 in PAM were shared between *corpus callosum* and *medulla oblongata*, 6 *versus* 15 pathways between *hippocampus* and *medulla oblongata*, and 5 *versus* 3 pathways between *corpus callosum* and *hippocampus*. Integrated Venn diagram analysis was performed using “the InteractiVenn”: a web-based tool for the analysis of complex data sets (Figs. 3*A*–3*B*) (46). See supplemental Data S3 for the listed differential pathways. Overexpressed proteins common to *medulla oblongata* and *hippocampus* (Fig. 3*A*) are involved in learning, epilepsy, neuronal activity and plasticity, neurotransmission and ischemia. For *hippocampus* and *corpus callosum* (Fig. 3*A*), the identified proteins are mainly involved in neurogenesis, cell proliferation and oxidative stress. For *medulla oblongata* and *corpus callosum* (Fig. 3*A*), the pattern is more related to cell damage and life span. The same analysis for unique pathways in *hippocampus* clearly showed protein patterns involved in neurogenesis, synaptogenesis, neurite outgrowth, neuroprotection, and axogenesis (Fig. 3*B*, supplemental Data S4). For *medulla oblongata* the proteins are mainly involved in pathways related to memory consolidation, epilepsy, cognition disorders, oligodendrocytes differentiation, amyotrophic lateral sclerosis, and spinocerebral ataxia type 1 (Fig. 3*B*). For *corpus callosum*, the proteins are mainly implicated in beta thalassemia, anemia and related hemoglobinopathies (Fig. 3*B*). All the results are in line with biological and physiological functions of these 3 brain regions.

##### PTM Analysis of Identified Proteins

**Fig. 4.**
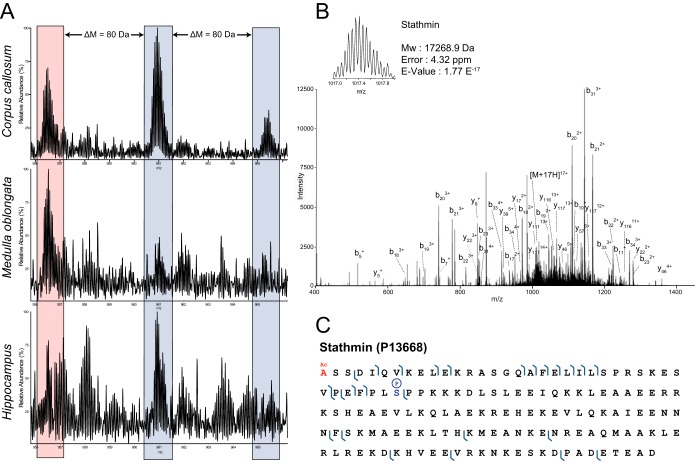
**ROI-specific PTM profile of Stathmin (*A*), which was identified in *corpus callosum* and *medulla oblongata* but not in *hippocampus*.** N-terminal acetylation is marked red, whereas the phosphorylations are marked blue. Precursor (z = 17) and assigned fragmentation spectrum of Nter-Acetylated and phosphorylated (Ser-38) Stathmin (*B*) and fragmentation map (*C*).

PTM analysis of proteins from the 3 regions revealed the presence of 91 proteins that were identified with PTMs, of which, 29 were detected in the *hippocampus*, 40 in the *corpus callosum* and 37 in the *medulla oblongata* (supplemental Data S2). Interestingly, some proteins show region-specific PTMs (Table I, supplemental Data S2). As an illustration, the most abundant PTM of stathmin in the *corpus callosum* (identified) and the *hippocampus* (detected but not identified) was the Nter-Acetyl + 1 Phosphorylation, whereas in the *medulla oblongata* (identified) it was the Nter-Acetylation (Fig. 4). Similarly, neurogranin was specifically phosphorylated in the *hippocampus*. Another example is the Astrocytic phosphoprotein (PEA-15), which was observed with a phosphorylated residue in the *corpus callosum* but not in the *medulla oblongata* (Table I and supplemental Data S2). Similarly, Parathymosin was identified with a mass shift of +79.94 Da in *hippocampus* by two spectra and with 5.89 and 5.38 ppm mass errors compared with theoretical mass plus a phosphorylation, thus implying a phosphorylated residue (Table I, Fig. 1*G* and supplemental Data S1 and 2). These data clearly revealed that the PTM state of proteins is linked to the brain regions where they are localized, and consequently with the biological function of the protein in relation to the physiological function of the considered brain region.

**Table I:** Region specific post-translationally modified proteins. PTMs from ProsightPC were concatenated with imputed PTMs from mass shifts (i.e. Acetylation (+42); Phosphorylation (+80))

Region	Accession number	PTM(s)	Protein name	Theo. mass (Da)	Obs. mass (Da)	Shift (Da)
***Corpus callosum***	P13668	N-acetyl-l-alanine, O-phospho-l-serine	Stathmin	17268.9	17269.0	0.094
	P31399	N-acetyl-l-alanine	ATP synthase subunit d, mitochondrial	18662.6	18662.6	0.082
	G3V9C0	N-acetyl-l-serine	Histone H2A	14037.9	14038.0	0.05
	Q5U318	N-acetyl-l-alanine, O-phospho-l-serine	Astrocytic phosphoprotein PEA-15	15021.7	15021.8	0.05
	D3ZHW9	N-acetyl-l-serine	Protein Shfm1	8183.53	8183.6	0.044
	B2RZ27	N-acetyl-l-serine	Protein Sh3bgrl3	10381.2	10381.3	0.033
	D3ZZW2	N-acetyl-l-serine	Protein LOC100910678	6972.9	6972.9	−0.008
	D3ZTB5	N-acetyl-l-alanine	Protein S100a13	11101.9	11101.0	−0.909
***Hippocampus***	Q04940	O-phospho-l-serine + Phosphorylation (+80)	Neurogranin	7440.43	7520.5	80.041
	M0R5I3	Phosphorylation (+80)	High mobility group nucleosomal binding domain 3, isoform CRA_a	10236.4	10316.4	79.973
	Q5U1W8	Phosphorylation (+80)	High-mobility group nucleosome binding domain 1	9987.3	10067.3	79.964
	P04550	N-acetyl-l-serine + Phosphorylation (+80)	Parathymosin	11463.2	11543.1	79.942
	P62329	N-acetyl-l-serine + Acetylation (+42)	Thymosin beta-4	4960.49	5002.5	42.029
***Medulla oblongata***	P06302	N-acetyl-l-serine + Acetylation (+42)	Prothymosin alpha	12286.1	12328.1	41.993
	P04631	N-acetyl-l-serine	Protein S100-B	10648	10648.1	0.05
	B2RYS2	N-acetyl-l-alanine	Cytochrome b-c1 complex subunit 7	13460.9	13460.9	0.047
	P63041	N-acetyl-l-methionine	Complexin-1	15154.5	15154.5	0.047
	P0CC09	N-acetyl-l-serine	Histone H2A type 2-A	13997.9	13997.9	0.042
	P02625	N-acetyl-l-serine	Parvalbumin alpha	11829	11829.0	0.032
	P11951	N-acetyl-l-serine	Cytochrome c oxidase subunit 6C-2	8360.42	8360.4	0.022
	Q5U318	N-acetyl-l-alanine	Astrocytic phosphoprotein PEA-15	14941.8	14940.8	−0.918
	P31044	N-acetyl-l-alanine	Phosphatidylethanolamine-binding protein 1	20699.4	20698.4	−0.935

##### Protein Fragments Linked to Brain Region Localization

Data analyses revealed the presence of protein fragments in the three brain regions (Table II and supplemental Data S8). These fragments are derived from large proteins such as neuropeptide precursors (somatostatin, proenkephalin, secretogranin 1 and 2), Synuclein (alpha, beta and gamma), Synaptosomal associated protein 25, DNA-(apurinic or apyrimidinic) protein (APEX), Hematological and neurological expressed 1 protein (HN1), Myelin basic protein (MBP) and Thymosin beta 4. The generated fragments are linked to the presence of processing enzymes *e.g.* pro-protein convertases, neutral endopeptidases, angiotensin-converting enzymes and aminopeptidases, which are differentially expressed in the brain region (38–41, 50, 51). Neuropeptide fragment precursors, neuromodulin and secretogranin 1 are principally detected in *hippocampus* whereas fragments of MBP and somatostatin are detected in majority in *medulla oblongata*. HN1 fragments are detected in *hippocampus*, whereas Secretogranin 2 is present in both *hippocampus* and *medulla oblongata*.

**Table II:** Most detected truncated protein. M.O: medulla oblongata; C.C: corpus callosum; Hi: hippocampus; AA: amino acids

Accession	Protein description	M.O	C.C	Hi	Detected length (AA)	Full length (AA)	Fragment (AA)	Fragment position
**P21571**	Chain [33–108] in ATP synthase-coupling factor 6, mitochondrial	√	√	√	76	108	33–108	C-terminal fragment
**P10818**	Chain [27–111] in Cytochrome c oxidase subunit 6A1, mitochondrial	√	√	√	85	111	27–111	C-terminal fragment
**P11240**	Chain [38–146] in Cytochrome c oxidase subunit 5A, mitochondrial	√	√	√	109	146	38–146	C-terminal fragment
**Q71UE8**	Chain [1–76] in NEDD8	√	√	√	76	81	1–76	N-terminal fragment
**P35171**	Chain [24–83] in Cytochrome c oxidase subunit 7A2, mitochondrial	√	√	√	60	83	24–83	C-terminal fragment
**P21571**	ATP synthase-coupling factor 6, mitochondrial	√	√	√	53	108	56–108	C-terminal fragment
**P47942**	Dihydropyrimidinase-related protein 2	√	√	√	55	572	518–572	C-terminal fragment
**Q63429**	Polyubiquitin-C	√	√		74	810	1–74	N-terminal fragment
**P28073**	Proteasome subunit beta type-6	√	√		17	238	78–94	Internal fragment
**P80432**	Chain [17–63] in Cytochrome c oxidase subunit 7C, mitochondrial	√	√	√	47	63	17–63	C-terminal fragment
**D4A5W9**	Synaptosomal-associated protein	√	√	√	45	206	162–206	C-terminal fragment
**P13668**	Stathmin		√		112	149	38–149	C-terminal fragment
**P21571**	ATP synthase-coupling factor 6, mitochondrial	√	√	√	75	108	34–108	C-terminal fragment
**F1LQ96**	Gamma-synuclein	√		√	30	122	93–122	C-terminal fragment
**F1LUV9**	Neural cell adhesion molecule 1		√		61	833	773–833	C-terminal fragment
**P37377**	Alpha-synuclein		√		73	140	68–140	C-terminal fragment
**O35314**	Secretogranin-1			√	30	675	292–321	Internal fragment
**P19527**	Neurofilament light polypeptide	√			74	542	469–542	C-terminal fragment
**Q5M7W5**	Microtubule-associated protein 4	√			16	1057	31–46	Internal fragment
**P26772**	10 kDa heat shock protein, mitochondrial	√	√		37	102	66–102	C-terminal fragment
**Q8R1R5**	CD99 antigen-like protein 2	√	√	√	24	246	223–246	C-terminal fragment
**Q6PCU8**	Chain [36–108] in NADH dehydrogenase [ubiquinone] flavoprotein 3, mitochondrial	√	√	√	73	108	36–108	C-terminal fragment
**F1LQ96**	Gamma-synuclein	√		√	48	122	75–122	C-terminal fragment

##### Alternative Protein Identification

Three alternative proteins were detected in spatially-resolved top-down proteomics experiments. AltCd3e and AltMyo1f were detected in *hippocampus* using LMJ and PAM, respectively, and AltGrb10 was detected in the *medulla oblongata* using PAM (Table III). These results suggest that the spatially-resolved proteomics strategy was suitable for studying the reference and hidden proteomes. We then enlarged this study by re-analyzing previous data obtained using whole rat brain sections (PMID:27512083) (42). Reanalysis of this dataset allowed the identification of 5 more alternative proteins (Table III, supplemental Data S6). These alternative proteins are translated from sequences located in mRNAs 3′UTR (AltSstr3, AltKcnq5, AltLdlr), 5′UTR regions (AltZbtb8a) of mRNAs and from a putative noncoding RNA (AltRn50_X_0580.1).

**Table III:** Alternative protein products identified by tissue top-down proteomics

Region	E-Value (P-score)	Observed Mass	Protein	Gene	AltORF localization on RNA	Transcript
*Hippocampus*	2.14 E-05 (3.80E-11)	4642.28	AltCd3e	Cd3e	3′UTR	ENSRNOT00000047291
	7.70E-05 (1.37E-10)	8154.94	AltMyo1f	Myo1f	CDS	ENSRNOT00000011513
*Medulla Oblongata*	1.06E-05 (1.89E-11)	15025.79	AltGrb10	Grb10	CDS	ENSRNOT00000085175
Whole brain section PMID 27512083	3.15E-05 (2.24E-10)	4760.46	AltRn50_X_05 80.1	Rn50_X_0580.1	ncRNA	ENSRNOT00000066392
	3.42E-05 (2.43E-10)	5000.62	AltSstr3	Sstr3	3′UTR	ENSRNOT00000009612
	4.23E-07 (7.52E-13)	3344.66	AltZbtb8a	Zbtb8a	5′UTR	ENSRNOT00000010983
	2.48E-09 (1.77E-14)	2825.44	AltKcnq5	Kcnq5	3′UTR	ENSRNOT00000040034
	1.36E-05 (9.68E-11)	4440.29	AltLdlr	Ldlr	3′UTR	ENSRNOT00000013496

##### Back Correlation to Localization by MALDI-MSI

**Fig. 5.**
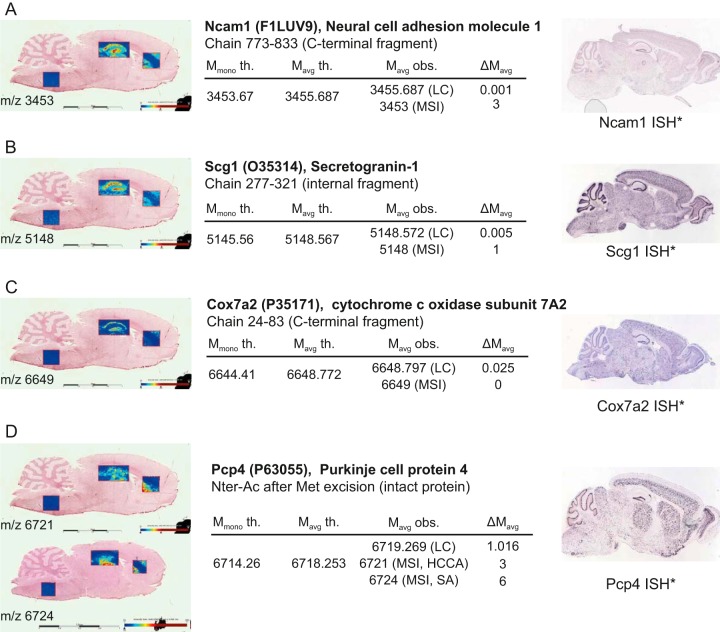
MALDI-MS images of top-down identified proteoforms of Ncam1 C-terminal fragment (*A*), Scg1 internal fragment (*B*), Cox7a2 C-terminal fragment (*C*) and intact Pcp4 N-terminally acetylated after initiator methionine excision (*D*) with their corresponding top-down (LC) and MSI *m*/*z* and their *in situ* hybridization images (ISH*). All values in tables are given in a.m.u. See also supplemental Data S7. *Image credit: Allen Institute (47).

Intact protein MSI experiments were performed to show ion distributions of the proteins identified by top-down MS. To this end, two images were acquired; the first section was prepared with HCCA/aniline matrix and the second one with SA/aniline. The images were acquired only on the three ROIs specified in the previous imaging experiment. Peaks obtained from these images were then matched with the M_avg_ derived from the top-down MS analysis performed on the entire rat brain tissue section. Thirty-five protein IDs obtained from the reference proteome were assigned to peaks obtained from both images with a ΔM_avgs_ cutoff ≤ 6 Da (Fig. 5*A*–5*D* and supplemental Data S7). This includes five proteins previously matched also with top-down MS data, namely PEP-19 (Pcp4, Fig. 5*D*), ubiquitin (Ubc), thymosin β-4 (Tmsb4x), thymosin β-10 (Tmsb10), and calmodulin (Calm1) (34).

**Fig. 6.**
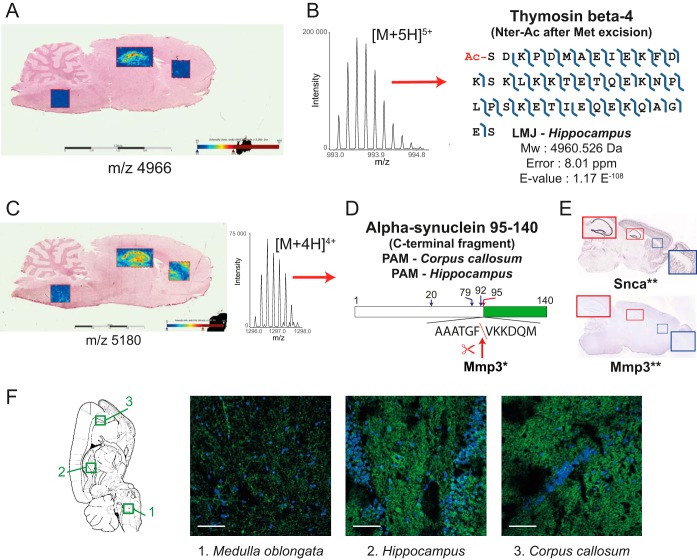
***A*, MALDI-MSI of m/z 4966 attributed to intact N-terminally acetylated form of Thymosin beta-4 with corresponding identification by spatially-resolved top-down proteomics (*B*).** *C*, MALDI-MSI of *m*/*z* 5180 attributed to C-terminal fragment of α-synuclein identified in *hippocampus* and *corpus callosum. D*, Schematic representation of protein fragment with predicted Stromelysin (Mmp3) cleavage sites (arrows and amino acid numbers) by PROSPER* (53) and identified form (red arrows). *E*, *In situ*- hybridization of α-synuclein (top) and Stromelysin (bottom). *F*, Tissue immunofluorescence image of α-synuclein using an antibody targeting the C-terminal of the protein. White scale bars = 50 μm. **Image credit: Allen Institute (47).

Fig. 6*A* shows the ion image of m/z 4966 assigned as the intact form (as hematopoietic system regulatory peptide) of thymosin β-4 (monoisotopic theoretical mass = 4960.49). The specific localization of *m*/*z* 4966 in the *hippocampus* can be clearly observed. Topdown data indicate that this isoform, detected as the [M+5H]^5+^ charge state, is the N-acetylated isoform after methionine excision (Fig. 6*B*). Its distribution in the *hippocampus* in MSI correlates well with the top-down data where this form was detected using PAM. Furthermore, its detection by MSI and assignment of N-acetylation by top-down is in accord with the MSI database reported by Maier *et al.* (52).

Fig. 6*C* shows the mapping of *m*/*z* 5180 assigned as the C-terminal fragment of α-synuclein (observed as the [M+4H]^4+^ charge state in topdown, Fig. 6*C*), showing its particularly intense distribution along the hippocampal dentate gyrus. Its distribution in the cerebral cortex observed in the ROI that includes the corpus callosum, was also detected in both MSI and spatially-resolved top-down proteomics. To verify the specific formation of this fragment, the putative protease cleavage sites found in the full amino acid sequence of α-synuclein was mapped using the PROtease Specificity Prediction servER (PROSPER, (53), https://prosper.erc.monash.edu.au), where it can be observed that cleavage by matrix metallopeptidase 3 (MMP3) can induce the generation of the C-terminal fragment (Fig. 6*D*). *In situ* hybridization of the genes that code for α-synuclein (Snca) and MMP3 in mouse brain obtained from the Allen Mouse Brain Atlas (http://mouse.brain-map.org/) (47) confirms the distribution of α-synuclein (strong) and MMP3 (weak) along the mouse hippocampal dentate gyrus (Fig. 6*E*). Localization of the α-synuclein was validated by tissue immunofluorescence showing strong signal in the *hippocampus* and *corpus callosum* and weak signal in the *medulla oblongata* (Fig. 6*F*).

In addition to the α-synuclein, the distribution of proteins GFAP, BASP1 and stathmin were further validated by IF experiments. supplemental Data S9 shows the confocal images after immunostaining against GFAP (red), showing highly positive astrocytes (GFAP^+++^) localized between the dentate gyrus and CA3 of Ammon's horn of the hippocampal formation. The signal is markedly absent in the corpus callosum and surrounding region (GFAP^−^), and is slightly present in the medulla oblongata region (GFAP^+/−^) as evidenced by immunoreactivity of several astrocyte processes projecting in different directions. The specific localization of GFAP-positive astrocytes in the hippocampus was confirmed after gathering z-series of images at varying focal planes throughout the entire tissue thickness of 10 μm. Likewise, GFAP was detected only in the hippocampus in MALDI-MSI and spatially-resolved top-down proteomics experiments. Results for the other proteins tested are shown in supplemental Data S9.

## DISCUSSION

We developed a novel strategy combining MALDI MS Imaging and spatially-resolved top-down proteomics to determine localized proteoforms, including truncated forms, fragments, and possibly altprots. First, molecular histology was performed using MALDI-MSI and spatial segmentation to distinguish ROIs within a tissue. These ROIs were then subjected to protein microextraction with ProteaseMAX rather than SDS or organic solvents. Protein microextraction efficiency was confirmed by nanoLC high resolution MS/MS analysis of rat brain tissue because we identified many proteins (123) compared with the 36 previously identified from a whole tissue proteomics study that performed extraction using acidified MeOH (34). Only 19 proteins were in common with those identified from this study. The 17 proteoforms absent in our study are small peptides less than 4500 Da and are more related to the neuropeptide family, *e.g.* chromogranin-A, cholecystokinin, proneuropeptide Y, secretogranin-2, proSAAS, cocaine- and amphetamine-regulated transcript protein, and oxysterol-binding protein, consistent with the brain regions selected in our study. Nevertheless, the common proteoforms identified are the same with the same PTMs.

It is interesting to note that LMJ and PAM do not identify the same proteins and are thus complementary, giving a total of 123 protein IDs overall. For example, somatostatin and peptide 143–185 of proenkephalin-A were specifically identified in LMJ samples whereas α-synuclein and neuromodulin where specifically identified in PAM experiments. Considering that the average size of brain cells is 15 μm and that we have microextracted 0.8 mm^2^ with LMJ and 1 mm^2^ with PAM, we estimate that we identified proteins from 4444 cells for LMJ and 5662 cells for PAM. By combining the two approaches, 15 specific and nonredundant proteins were identified from the corpus callosum, 17 from the *medulla oblongata* and 24 from the *hippocampus* (Tables I and II). 35 are common to the 3 brain regions, 16 between *corpus callosum* and *medulla oblongata*, 8 between *corpus callosum* and *hippocampus* and 8 between *medulla oblongata* and *hippocampus*. Proteins identified with PAM are mainly present in the cytoplasm (62%), mitochondrial membrane (9.3%) or organelles and plasma membranes (28.7%). With LMJ, the proteins identified are from organelles (51.5%) and the cytoplasm (47.7%).

These studies performed by spatially-resolved top-down proteomics are in line and complementary to our previous studies based on spatially-resolved bottom-up proteomics (1, 17, 27) as it gives information about the precursor mass and PTMs detectable by measuring the ΔM(s) between the intact precursor within a close retention time window. Indeed, our approach successfully discriminate stathmin PTMs between different regions of rat brain tissue (Fig. 4). We showed that stathmin is more abundant in *corpus callosum* and *medulla oblongata* and its PTM pattern is specific for each of these two regions. The ratio phospho-stathmin/Nter-Ac was significantly higher in the *corpus callosum*, suggesting a different biological activity in these two regions of the brain (Fig. 4). Similarly, out of the 41 unique proteins that were identified with PTMs (supplemental Data S1 and 2), 22 had region specific PTMs (Table I). The most prevalent PTMs are the N-acetylation of proteins and phosphorylation. For example, we found that α-synuclein presents one PTM, *i.e.* N-acetyl-l-methionine in *medulla oblongata*, *hippocampus* and *corpus callosum*. In literature it has been shown that α-synuclein acetylation at Met in position 1 seems to be important for its proper folding (54)(55). Similarly, the Astrocytic phosphoprotein (PEA-15) possesses N-acetyl-l-alanine in *medulla oblongata* and N-acetyl-l-alanine plus O-phospho-l-serine in *corpus callosum*. None of them have been previously identified (56).

In the same way, we identified protein fragments from proteins with distribution and presented a specific cleavage form across each brain region. Majority of the identified fragments are large neuropeptides like synenkephalin and secretogranins 1 and 2. These fragments are produced by enzymatic cleavage of the pro-protein convertase family like PC1/3, PC2 or PC5, PACE4 (38). We previously demonstrated the role of these enzymes in proenkephalin maturation (51, 57) and found some of these neuropeptide fragments in temporal lobe epilepsy (58) and Alzheimer's disease (59), such as secretogranins for example. Synenkephalin is implicated in circadian rhythm in the *hippocampus* (60), Snap25 is implicated in synaptogenesis and memory consolidation (61–63). As previously demonstrated, we confirmed that the somatostatin is present in *medulla oblongata* (64) whereas we showed for the first time the presence of the hematological and neurological expressed 1 protein in the *hippocampus* (fragment) and *corpus callosum* (full length after methionine excision).

Besides these novel protein fragments, another small family of proteins has been identified from the hidden proteome. In fact, more and more evidence suggests that mRNAs contain more than one coding sequence and could be translated into an annotated or reference protein and at least one alternative protein (36, 37). We tested if our strategy was able to detect intact alternative proteins. We identified 3 alternative proteins (Table III) by the spatially-resolved top-down proteomics approach that share no sequence similarity with annotated *rattus norvegicus* proteins. Of the 5 novel altprots identified by reanalysis of the study on whole tissue sections (Alt-Kcnq5, Alt-Zbtb8a, Alt-Sstr3, Alt-Ldlr and a noncoding RNA Alt-Rn50_X_0580.1), 3 of them are receptors as reference proteins *i.e.* somatostatin 3 receptor, potassium voltage-gated channel subfamily Q member 5, and low-density lipoprotein receptor. It is interesting to note that these 3 receptors are known to be expressed in *hippocampus* specifically (65–67).

Back correlation of spatially-resolved top-down proteomics protein IDs with MALDI MS images allowed to localize 35 identified proteins (Fig. 5 and supplemental Data S7). The correlation included proteins with PTMs or enzymatic cleavage whose distribution varies differently in the 3 regions in line with identified biological processes taking place in each individual region. As an example, the truncated, N-acetylated form of thymosin β-4 was mapped in MSI and its distribution was compared with the result of the top-down data, showing good correlation of the results from the two approaches (Fig. 6*A* and 6*B*). The C-terminal fragment of α-synuclein likewise showed very good correlation of results (Fig. 6*C*). More importantly, the distribution of this fragment in the hippocampal dentate gyrus in MSI can be correlated with the abundance of α-synuclein and MMP3 in the same region in ISH experiments on mouse brain. MMP3 can cleave α-synuclein at F^94^, yielding the natively unstructured C-terminal fragment aa 95–140 (5.74 kDa) (Fig. 6*D* and 6*E*). Tissue immunofluorescence validated α-synuclein's localization showing strong signal in *hippocampus* and *corpus callosum* indicating the presence of the protein in these regions (Fig. 6*F*). However, MALDI-MSI revealed that the C-terminal fragment has a strong and precise tissue localization in the hippocampal dentate gyrus and moderately around the *corpus callosum*, matching the MMP3 *in situ* hybridization (47). This result exposes the great capabilities of spatially-resolved top-down proteomics associated to MALDI-MSI to detect and localize truncated proteoforms that can be challenging using antibody-based tissue characterization methods. Other MMP3-produced C-terminally truncated peptides of α-synuclein (aa 1–78, 1–91 and 1–93) have been reported under stress conditions, with aa 1–93 being implicated in dopamine neuronal loss in *substantia nigra*, suggesting that overexpression of the fragments could have a significant impact in Parkinson's disease (68). What role aa 95–140 has in this regard thus needs to be further investigated.

Taken together, our results show that spatially-resolved top-down proteomics linked to MALDI-MSI can be used to search for biomarkers, PTM detection and to identify novel proteins expressed from altORFs.

## DATA AVAILABILITY

The mass spectrometry proteomics data have been deposited to the ProteomeXchange Consortium via the PRIDE (43) partner repository with the data set identifier PXD005424.

## Supplementary Material

Supplemental Data

## Footnotes

^1^ The abbreviations used are:

MSI: mass spectrometry imaging
LMJ: liquid microjunction
PAM: paraffin-assisted microdissection
PTM: post-translational modification
CDS: coding DNA sequence
altORF: alternative open reading frame
ROI: regions of interest.

## References

[1] Quanico J., Franck J., Dauly C., et al (2013) Development of liquid microjunction extraction strategy for improving protein identification from tissue sections. J. Proteomics 79, 200–218 doi: 10.1016/j.jprot.2012.11.025 23291530

[2] Viale G., Slaets L., de Snoo F. A., et al (2016) Discordant assessment of tumor biomarkers by histopathological and molecular assays in the EORTC randomized controlled 10041/BIG 03–04 MINDACT trial breast cancer : Intratumoral heterogeneity and DCIS or normal tissue components are unlikely to be the cau. Breast Cancer Res. Treat 155, 463–469 doi: 10.1007/s10549-016-3690-6 26820652 PMC4764628

[3] Massard C., Oulhen M., Le Moulec S., et al (2016) Phenotypic and genetic heterogeneity of tumor tissue and circulating tumor cells in patients with metastatic castration-resistant prostate cancer: A report from the PETRUS prospective study. Oncotarget 7, 55069–55082 doi: 10.18632/oncotarget.10396 27391263 PMC5342402

[4] Krönig M., Walter M., Drendel V., et al (2015) Cell type specific gene expression analysis of prostate needle biopsies resolves tumor tissue heterogeneity. Oncotarget 6, 1302–1314 doi: 10.18632/oncotarget.2744 25514598 PMC4359234

[5] Johann D. J., Rodriguez-Canales J., Mukherjee S., et al (2009) Approaching solid tumor heterogeneity on a cellular basis by tissue proteomics using laser capture microdissection and biological mass spectrometry. J. Proteome Res. 8, 2310–2318 doi: 10.1021/pr8009403 19284784 PMC2858576

[6] Johann D. J., Mukherjee S., Prieto D. A., et al (2011) Profiling solid tumor heterogeneity by LCM and biological MS of fresh-frozen tissue sections. Methods Mol. Biol. 755, 95–106 doi: 10.1007/978-1-61779-163-5_8 21761297

[7] Sugihara Y., Taniguchi H., Kushima R., et al (2013) Laser microdissection and two-dimensional difference gel electrophoresis reveal proteomic intra-tumor heterogeneity in colorectal cancer. J. Proteomics 78, 134–147. doi: 10.1016/j.jprot.2012.11.009 23178874

[8] Celis J. E., Celis P., Palsdottir H., et al (2002) Proteomic strategies to reveal tumor heterogeneity among urothelial papillomas. Mol. Cell. Proteomics 1, 269–279 12096109 10.1074/mcp.m100031-mcp200

[9] Bonnel D., Longuespee R., Franck J., et al (2011) Multivariate analyses for biomarkers hunting and validation through on-tissue bottom-up or in-source decay in MALDI-MSI: application to prostate cancer. Anal. Bioanal. Chem. 401, 149–165 21519967 10.1007/s00216-011-5020-5

[10] Bruand J., Alexandrov T., Sistla S., et al (2011) AMASS: Algorithm for MSI analysis by semi-supervised segmentation. J. Proteome Res. 10, 4734–4743 doi: 10.1021/pr2005378 21800894 PMC3190602

[11] Wisztorski M., Desmons A., Quanico J., et al (2016) Spatially-resolved protein surface microsampling from tissue sections using liquid extraction surface analysis. Proteomics 16, 1622–1632 doi: 10.1002/pmic.201500508 26929135

[12] Wisztorski M., Fatou B., Franck J., et al (2013) Microproteomics by liquid extraction surface analysis: application to FFPE tissue to study the fimbria region of tubo-ovarian cancer. Proteomics Clin. Appl. 7, 234–240 doi: 10.1002/prca.201200070 23339084

[13] Walworth M. J., Stankovich J. J., Van Berkel G. J., et al (2011) Hydrophobic treatment enabling analysis of wettable surfaces using a liquid microjunction surface sampling probe/electrospray ionization-mass spectrometry system. Anal. Chem. 83, 591–597 doi: 10.1021/ac102634e 21158402

[14] Walworth M. J., ElNaggar M. S., Stankovich J. J., et al (2011) Direct sampling and analysis from solid-phase extraction cards using an automated liquid extraction surface analysis nanoelectrospray mass spectrometry system. Rapid Commun. Mass Spectrom. 25, 2389–2396 doi: 10.1002/rcm.5132 21793068

[15] Van Berkel G. J., and Kertesz V. (2013) Continuous-flow liquid microjunction surface sampling probe connected on-line with high-performance liquid chromatography/mass spectrometry for spatially resolved analysis of small molecules and proteins. Rapid Commun. Mass Spectrom. 27, 1329–1334 doi: 10.1002/rcm.6580 23681810

[16] Van Berkel G. J., and Kertesz V. (2009) Application of a liquid extraction based sealing surface sampling probe for mass spectrometric analysis of dried blood spots and mouse whole-body thin tissue sections. Anal. Chem. 81, 9146–9152 doi: 10.1021/ac901712b 19817477

[17] Franck J., Quanico J., Wisztorski M., et al (2013) Quantification-based mass spectrometry imaging of proteins by parafilm assisted microdissection. Anal. Chem. 85, 8127–8134 doi: 10.1021/ac4009397 23869483

[18] Kertesz V., Weiskittel T. M., and Van Berkel G. J. (2015) An enhanced droplet-based liquid microjunction surface sampling system coupled with HPLC-ESI-MS/MS for spatially resolved analysis. Anal. Bioanal. Chem. 407, 2117–2125. doi: 10.1007/s00216-014-8287-5 25377777

[19] Kertesz V., and Van Berkel G. J. (2014) Sampling reliability, spatial resolution, spatial precision, and extraction efficiency in droplet-based liquid microjunction surface sampling. Rapid Commun. Mass Spectrom. 28, 1553–1560 doi: 10.1002/rcm.6931 24861607

[20] Kertesz V., and Van Berkel G. J. (2013) Automated liquid microjunction surface sampling-HPLC-MS/MS analysis of drugs and metabolites in whole-body thin tissue sections. Bioanalysis 5, 819–826 doi: 10.4155/bio.13.42 23534426

[21] Kertesz V., and Van Berkel G. J. (2010) Fully automated liquid extraction-based surface sampling and ionization using a chip-based robotic nanoelectrospray platform. J. Mass Spectrom. 45, 252–260 doi: 10.1002/jms.1709 20020414

[22] Kertesz V., and Van Berkel G. J. (2010) Liquid microjunction surface sampling coupled with high-pressure liquid chromatography-electrospray ionization-mass spectrometry for analysis of drugs and metabolites in whole-body thin tissue sections. Anal. Chem. 82, 5917–5921 doi: 10.1021/ac100954p 20560529

[23] Emory J. F., Walworth M. J., Van Berkel G. J., et al (2010) Direct analysis of reversed-phase high-performance thin layer chromatography separated tryptic protein digests using a liquid microjunction surface sampling probe/electrospray ionization mass spectrometry system. Eur. J. Mass Spectrom. 16, 21–33 doi: 10.1255/ejms.1041 20065522

[24] Elnaggar M. S., Barbier C., and Van Berkel G. J. (2011) Liquid microjunction surface sampling probe fluid dynamics: computational and experimental analysis of coaxial intercapillary positioning effects on sample manipulation. J. Am. Soc. Mass Spectrom. 22, 1157–1166 doi: 10.1007/s13361-011-0145-5 21953098

[25] Quanico J., Franck J., Cardon T., et al (2016) NanoLC-MS coupling of liquid microjunction microextraction for on-tissue proteomic analysis. Biochim. Biophys. Acta - Proteins Proteomics doi: 10.1016/j.bbapap.2016.11.002 27836619

[26] Zimmerman T. A., Rubakhin S. S., Sweedler J V. (2011) MALDI mass spectrometry imaging of neuronal cell cultures. J. Am. Soc. Mass Spectrom. 22, 828–836 doi: 10.1007/s13361-011-0111-2 21472517 PMC3113696

[27] Quanico J., Franck J., Gimeno J. P., et al (2015) Parafilm-assisted microdissection: a sampling method for mass spectrometry-based identification of differentially expressed prostate cancer protein biomarkers. Chem. Commun. 51, 4564–4567 doi: 10.1039/C4CC08331H 25490716

[28] Nicolardi S., Switzar L., Deelder A. M., et al (2015) Top-down MALDI-in-source decay-FTICR mass spectrometry of isotopically resolved proteins. Anal. Chem. 87, 3429–3437 doi: 10.1021/ac504708y 25719938

[29] Kou Q., Zhu B., Wu S., et al (2016) Characterization of proteoforms with unknown post-translational modifications using the MIScore. J. Proteome Res. 15, 2422–2432 doi: 10.1021/acs.jproteome.5b01098 27291504 PMC5359983

[30] Fellers R. T., Greer J. B., Early B. P., et al (2015) ProSight Lite: graphical software to analyze top-down mass spectrometry data. Proteomics 15, 1235–1238 doi: 10.1002/pmic.201570050 25828799 PMC4445472

[31] Birner-Gruenberger R., and Breinbauer R. (2015) Weighing the proteasome for covalent modifications. Chem. Biol. 22, 315–316 doi: 10.1016/j.chembiol.2015.03.003 25794435

[32] Tran J. C., Zamdborg L., Ahlf D. R., et al (2011) Mapping intact protein isoforms in discovery mode using top-down proteomics. Nature 480, 254–258 doi: 10.1038/nature10575 22037311 PMC3237778

[33] Liu X., Hengel S., Wu S., et al (2013) Identification of ultramodified proteins using top-down tandem mass spectra. 10.1021/pr400849y PMC3905687 24188097

[34] Ye H., Mandal R., Catherman A., et al (2014) Top-down proteomics with mass spectrometry imaging: a pilot study towards discovery of biomarkers for neurodevelopmental disorders. PLoS ONE 9, e92831 doi: 10.1371/journal.pone.0092831 24710523 PMC3978070

[35] Laouirem S., Le Faouder J., Alexandrov T., et al (2014) Progression from cirrhosis to cancer is associated with early ubiquitin post-translational modifications: identification of new biomarkers of cirrhosis at risk of malignancy. J. Pathol. 234, 452–463 doi: 10.1002/path.4398 24979321

[36] Vanderperre B., Lucier J.-F., Bissonnette C., et al (2013) Direct detection of alternative open reading frames translation products in human significantly expands the proteome. PLoS ONE 8, e70698 doi: 10.1371/journal.pone.0070698 23950983 PMC3741303

[37] Mouilleron H., Delcourt V., and Roucou X. (2016) Death of a dogma: eukaryotic mRNAs can code for more than one protein. Nucleic Acids Res. 44:14–23 doi: 10.1093/nar/gkv1218 26578573 PMC4705651

[38] Zheng M., Seidah N. G., and Pintar J. E. (1997) The developmental expression in the rat CNS and peripheral tissues of proteases PC5 and PACE4 mRNAs: comparison with other proprotein processing enzymes. Dev. Biol. 181, 268–283 doi: 10.1006/dbio.1996.8402 9013936

[39] Walther T., Albrecht D., Becker M., et al (2009) Improved learning and memory in aged mice deficient in amyloid beta-degrading neutral endopeptidase. PLoS ONE 4, e4590 doi: 10.1371/journal.pone.0004590 19240795 PMC2643003

[40] Saavedra J. M., Fernandez-Pardal J., and Chevillard C. (1982) Angiotensin-converting enzyme in discrete areas of the rat forebrain and pituitary gland. Brain Res 245, 317–325 6289966 10.1016/0006-8993(82)90814-9

[41] Harmer D., Gilbert M., Borman R., and Clark K. L. (2002) Quantitative mRNA expression profiling of ACE 2, a novel homologue of angiotensin converting enzyme. FEBS Lett. 532, 107–110 12459472 10.1016/s0014-5793(02)03640-2

[42] Quanico J., Franck J., Salzet M., and Fournier I. (2016) On-tissue direct monitoring of global hydrogen/deuterium exchange by MALDI mass spectrometry: TDXMS. Mol. Cell. Proteomics 33, 0–3 doi: 10.1074/mcp.O116.059832 PMC5054352 27512083

[43] Vizcaino J. A., Csordas A., Del-Toro N., et al (2016) 2016 update of the PRIDE database and its related tools. Nucleic Acids Res. 44, D447–D456 doi: 10.1093/nar/gkv1145 26527722 PMC4702828

[44] Bonnet A., Lagarrigue S., Liaubet L., et al (2009) Pathway results from the chicken data set using GOTM, Pathway Studio and Ingenuity softwares. BMC Proc. 3, S11. doi: 10.1186/1753-6561-3-S4-S11 PMC2712741 19615111

[45] Yuryev A., Kotelnikova E., and Daraselia N. (2009) Ariadne's ChemEffect and pathway studio knowledge base. Expert Opin Drug Discov 4, 1307–1318 doi: 10.1517/17460440903413488 23480468

[46] Heberle H., Meirelles G. V., da Silva F. R., et al (2015) InteractiVenn: a web-based tool for the analysis of sets through Venn diagrams. BMC Bioinformatics 16, 169 doi: 10.1186/s12859-015-0611-3 25994840 PMC4455604

[47] Lein E. S., Hawrylycz M. J., Ao N., et al (2007) Genome-wide atlas of gene expression in the adult mouse brain. Nature 445, 168–176 doi: 10.1038/nature05453 17151600

[48] Chick J. M., Kolippakkam D., Nusinow D. P., et al (2015) A mass-tolerant database search identifies a large proportion of unassigned spectra in shotgun proteomics as modified peptides. Nat. Biotechnol. doi: 10.1038/nbt.3267 PMC4515955 26076430

[49] Szklarczyk D., Franceschini A., Wyder S., et al (2015) STRING v10: protein-protein interaction networks, integrated over the tree of life. Nucleic Acids Res. 43, D447–D452 doi: 10.1093/nar/gku1003 25352553 PMC4383874

[50] Salzet M., Vieau D., and Day R. (2000) Crosstalk between nervous and immune systems through the animal kingdom: focus on opioids. Trends Neurosci. 23, 550–555 11074264 10.1016/s0166-2236(00)01642-8

[51] Day R, Salzet M The neuroendocrine phenotype, cellular plasticity, and the search for genetic switches: redefining the diffuse neuroendocrine system. Neuro. Endocrinol. Lett. 23, 447–451 12500170

[52] Maier S. K., Hahne H., Gholami A. M., et al (2013) Comprehensive identification of proteins from MALDI imaging. Mol. Cell. Proteomics 12, 2901–2910 doi: 10.1074/mcp.M113.027599 23782541 PMC3790299

[53] Song J., Tan H., Perry A. J., et al (2012) PROSPER: an integrated feature-based tool for predicting protease substrate cleavage sites. PLoS ONE doi: 10.1371/journal.pone.0050300 PMC3510211 23209700

[54] Trexler A. J., and Rhoades E. (2012) N-Terminal acetylation is critical for forming α-helical oligomer of α-synuclein. Protein Sci. 21, 601–605 doi: 10.1002/pro.2056 22407793 PMC3403458

[55] Sarafian T. A., Ryan C. M., Souda P., et al (2013) Impairment of mitochondria in adult mouse brain overexpressing predominantly full-length, N-terminally acetylated human ??-synuclein. PLoS ONE doi: 10.1371/journal.pone.0063557 PMC3646806 23667637

[56] Lundby A., Secher A., Lage K., et al (2012) Quantitative maps of protein phosphorylation sites across 14 different rat organs and tissues. Nat. Commun. 3, 876 doi: 10.1038/ncomms1871 22673903 PMC3621391

[57] Salzet M. (2001) Neuroimmunology of opioids from invertebrates to human. Neuro. Endocrinol. Lett. 22, 467–474 11781546

[58] Mériaux C., Franck J., Park D. B., et al (2014) Human temporal lobe epilepsy analyses by tissue proteomics. Hippocampus 24, 628–642 doi: 10.1002/hipo.22246 24449190

[59] Ho Kim J., Franck J., Kang T., et al (2015) Proteome-wide characterization of signalling interactions in the hippocampal CA4/DG subfield of patients with Alzheimer's disease. Sci. Rep. 5, 11138 doi: 10.1038/srep11138 26059363 PMC4462342

[60] Miguel Asai M. A., Lilian Mayagoitia L. M., David García D. G., et al (2007) Rat brain opioid peptides-circadian rhythm is under control of melatonin. Neuropeptides 41, 389–397 doi: 10.1016/j.npep.2007.09.001 17988732

[61] Hou Q., Gao X., Zhang X., et al (2004) SNAP-25 in hippocampal CA1 region is involved in memory consolidation. Eur. J. Neurosci. 20, 1593–1603 doi: 10.1111/j.1460-9568.2004.03600.x 15355326

[62] Song H., Stevens C. F., and Gage F. H. (2002) Neural stem cells from adult hippocampus develop essential properties of functional CNS neurons. Nat. Neurosci. 5, 438–445. doi: 10.1038/nn844 11953752

[63] Aigner L., Arber S., Kapfhammer J. P., et al (1995) Overexpression of the neural growth-associated protein GAP-43 induces nerve sprouting in the adult nervous system of transgenic mice. Cell 83, 269–278 7585944 10.1016/0092-8674(95)90168-x

[64] Johansson O., Hökfelt T., and Elde R. P. (1984) Immunohistochemical distribution of somatostatin-like immunoreactivity in the central nervous system of the adult rat. Neuroscience 13, 265–339 6514182 10.1016/0306-4522(84)90233-1

[65] Dournaud P., Jazat-Poindessous F., Slama A., et al (1996) Correlations between water maze performance and cortical somatostatin mRNA and high-affinity binding sites during ageing in rats. Eur. J. Neurosci. 8, 476–485 8963438 10.1111/j.1460-9568.1996.tb01231.x

[66] Yus-Nájera E., Muñoz A., Salvador N., et al (2003) Localization of KCNQ5 in the normal and epileptic human temporal neocortex and hippocampal formation. Neuroscience 120, 353–364 12890507 10.1016/s0306-4522(03)00321-x

[67] Poirier J., Baccichet A., Dea D., and Gauthier S. (1993) Cholesterol synthesis and lipoprotein reuptake during synaptic remodelling in hippocampus in adult rats. Neuroscience 55, 81–90 8350994 10.1016/0306-4522(93)90456-p

[68] Choi D. H., Kim Y. J., Kim Y. G., et al (2011) Role of matrix metalloproteinase 3-mediated α-synuclein cleavage in dopaminergic cell death. J. Biol. Chem. 286, 14168–14177 doi: 10.1074/jbc.M111.222430 21330369 PMC3077618

