# Supplementary material for: Spatially-Resolved Top-down Proteomics Bridged to MALDI MS Imaging Reveals the Molecular Physiome of Brain Regions
Source: Mol Cell Proteomics. 2017 Nov 9;17(2):357–72. doi: 10.1074/mcp.M116.065755 (PMC5795397; doi:10.1074/mcp.M116.065755)
Supplement: Supplemental Data [file supp_17_2_357__index.html]

Spatially-Resolved Top-down Proteomics Bridged to MALDI MS Imaging Reveals the Molecular Physiome of Brain Regions — On-tissue Spatially-Resolved Top-down Proteomics Bridged to MALDI-MSI — Supplemental Data 

# Spatially-Resolved Top-down Proteomics Bridged to MALDI MS Imaging Reveals the Molecular Physiome of Brain Regions

## Supplemental Data

- Supplementary data Legend (.docx, 12 KB) - Supplementary data Legend
- Supplementary data 1 (.xlsx, 361 KB) - Filtered ProSightPC identification table.
- Supplementary data 2 (.xlsx, 46 KB) - List of identified proteins by top-down MS using liquid microjunction (LMJ) microextraction and parafilm-assisted microdissection (PAM).
- Supplementary data 3 (.xlsx, 17 KB) - Neighboring pathways shared between 2 regions of the rat brain (hippocampus and corpus callosum, hippocampus and medulla oblongata and corpus callosum and medulla oblongata).
- Supplementary data 4 (.xlsx, 99 KB) - Rat brain region-specific neighboring pathways (hippocampus, medulla oblongata and corpus callosum).
- Supplementary data 5 (.xlsx, 56 KB) - Rat brain shared neighboring pathways (hippocampus, medulla oblongata and corpus callosum).
- Supplementary data 6 (.xlsx, 153 KB) - Identified alternative proteins by top-down from rat brain microproteomics and whole tissue section (PMID 27512083)
- Supplementary data 7 (.pptx, 51.1 MB) - Assignment of MALDI MSI m/z intervals using protein identifications obtained by top-down MS.
- Supplementary data 8 (.xlsx, 16 KB) - List of truncated proteins identified via top-down microproteomics.
- Supplementary data 9 (.pptx, 7.1 MB) - Supplementary Data 9: Tissue immunofluorescence experiments.
