# Supplementary material for: Spatially-Resolved Top-down Proteomics Bridged to MALDI MS Imaging Reveals the Molecular Physiome of Brain Regions
Source: Mol Cell Proteomics. 2017 Nov 9;17(2):357–72. doi: 10.1074/mcp.M116.065755 (PMC5795397; doi:10.1074/mcp.M116.065755)
Supplement: Supplemental Data [file 10.1074_M116.065755_mcp.M116.065755-1.docx]

**SUPPLEMENTARY DATA**

**Supplementary Data 1**: Filtered ProSightPC identification table.

**Supplementary Data 2**: List of identified proteins by spatially-resolved top-down MS using liquid microjunction (LMJ) microextraction and parafilm-assisted microdissection (PAM).

**Supplementary Data 3**: Neighboring pathways shared between 2 regions of the rat brain (*hippocampus* and *corpus* *callosum*, *hippocampus* and *medulla* *oblongata* and *corpus* *callosum* and *medulla* *oblongata*).

**Supplementary Data 4**: Rat brain region-specific neighboring pathways (*hippocampus*, *medulla* *oblongata* and *corpus* *callosum*).

**Supplementary Data 5:** Rat brain shared neighboring pathways (*hippocampus*, *medulla* *oblongata* and *corpus* *callosum*).

**Supplementary Data 6:** Identified alternative proteins by top-down from rat brain spatially-resolved and whole tissue section proteomics (PMID 27512083).

**Supplementary Data 7:** Assignment of MALDI-MSI m/z intervals using protein identifications obtained by top-down MS.

**Supplementary Data 8:** List of truncated proteins identified *via* spatially-resolved top-down proteomics

**Supplementary Data 9:** Tissue immunofluorescence experiments.
