# Supplementary material for: Spatially-Resolved Top-down Proteomics Bridged to MALDI MS Imaging Reveals the Molecular Physiome of Brain Regions
Source: Mol Cell Proteomics. 2017 Nov 9;17(2):357–72. doi: 10.1074/mcp.M116.065755 (PMC5795397; doi:10.1074/mcp.M116.065755)
Supplement: Supplemental Data [file 10.1074_M116.065755_mcp.M116.065755-10.pptx]

## Slide 1
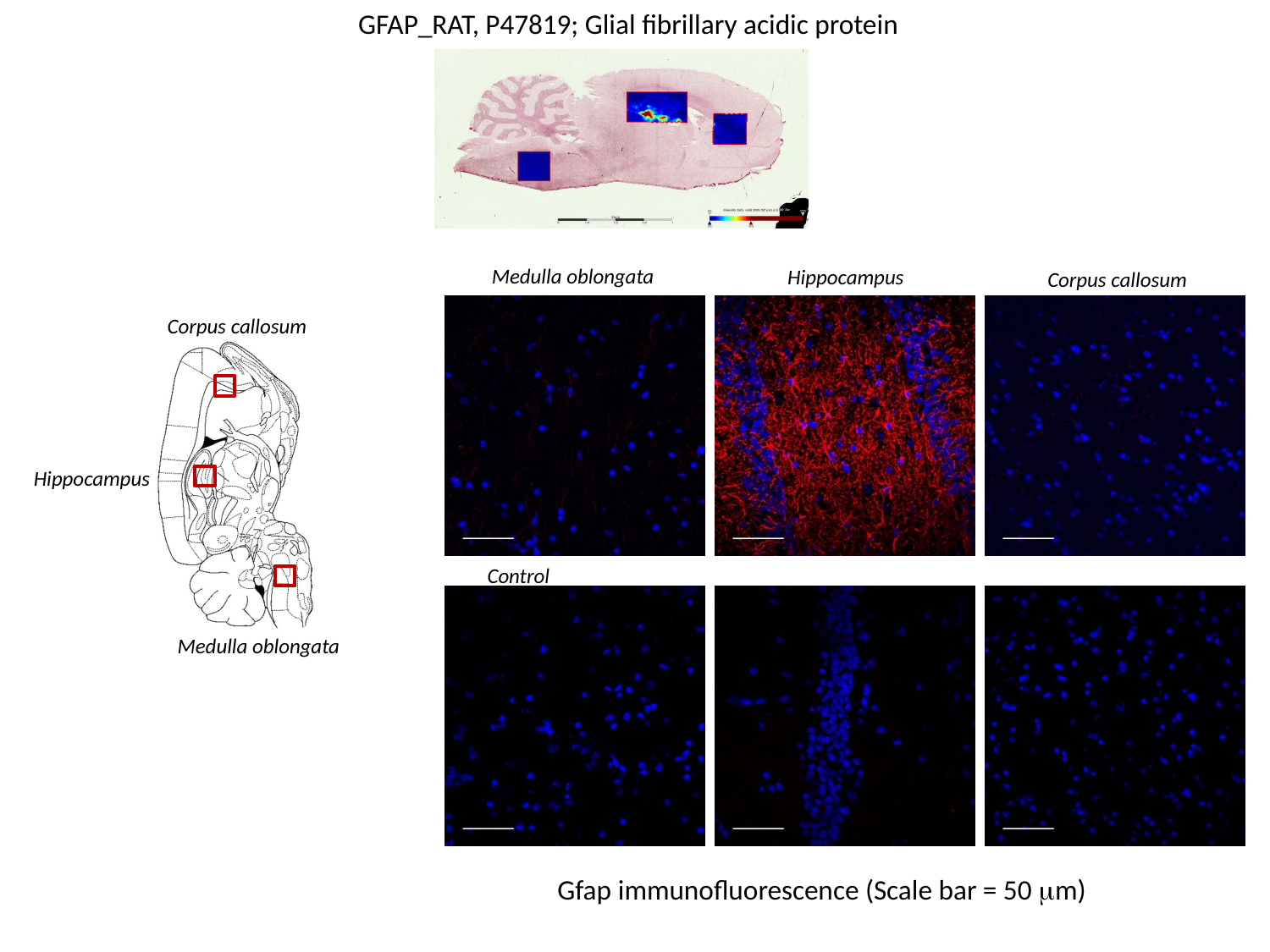

GFAP_RAT, P47819; Glial fibrillary acidic protein
Medulla oblongata
Hippocampus
Corpus callosum
Corpus callosum
Hippocampus
Control
Medulla oblongata
Gfap immunofluorescence (Scale bar = 50 μm)

## Slide 2
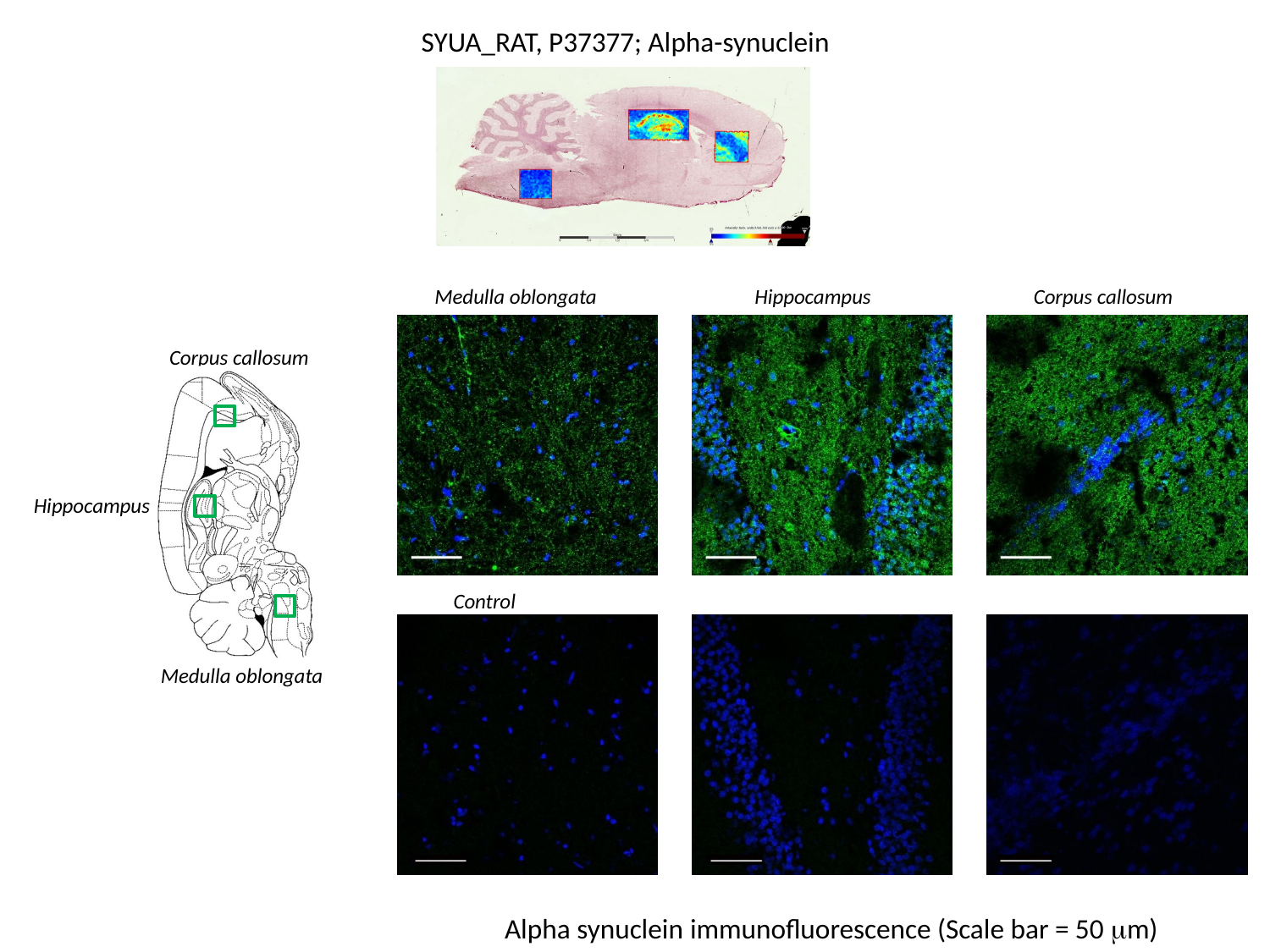

SYUA_RAT, P37377; Alpha-synuclein
Medulla oblongata
Hippocampus
Corpus callosum
Corpus callosum
Hippocampus
Control
Medulla oblongata
Alpha synuclein immunofluorescence (Scale bar = 50 μm)

## Slide 3
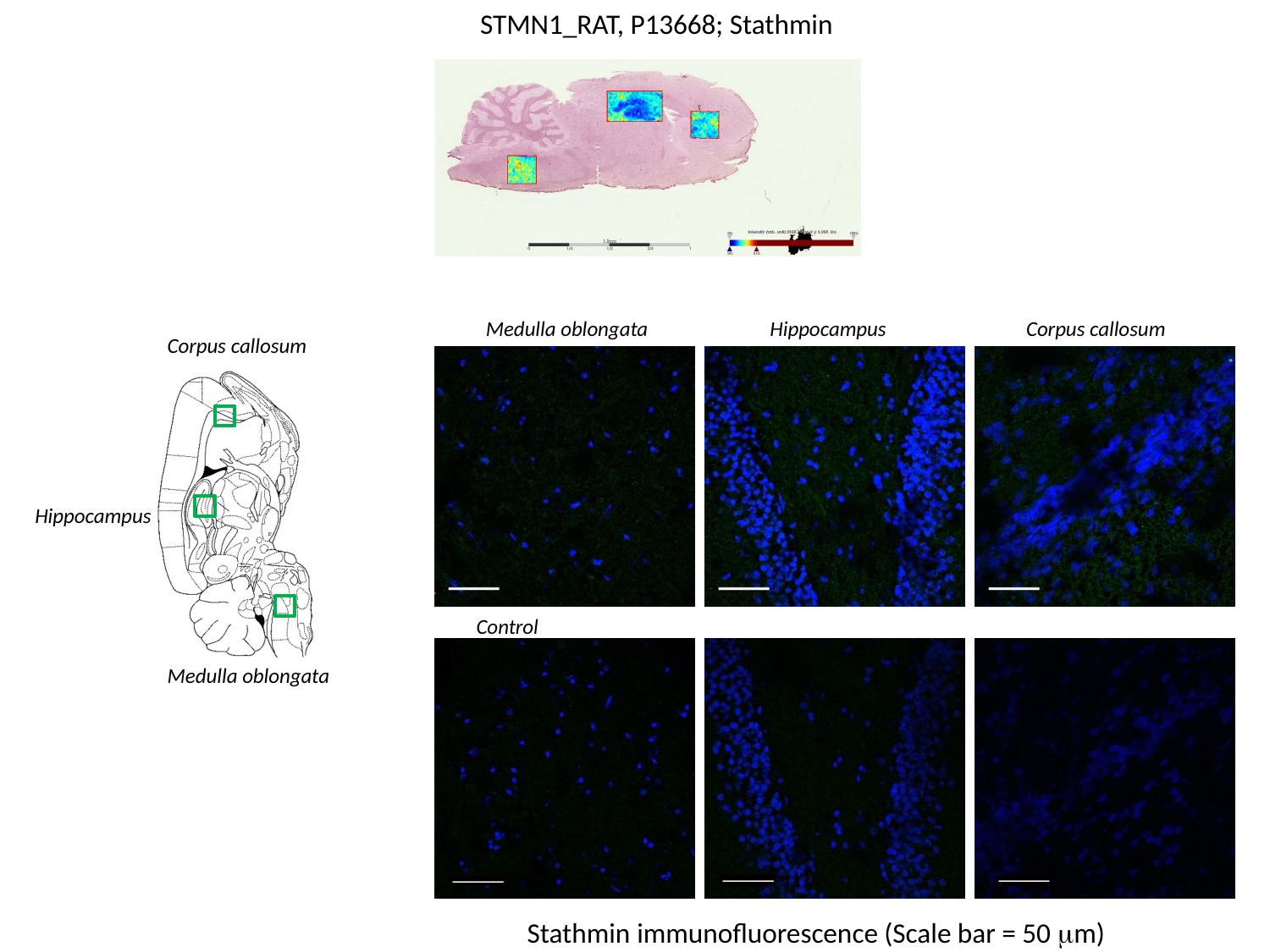

STMN1_RAT, P13668; Stathmin
Corpus callosum
Medulla oblongata
Hippocampus
Corpus callosum
Hippocampus
Control
Medulla oblongata
Stathmin immunofluorescence (Scale bar = 50 μm)

## Slide 4
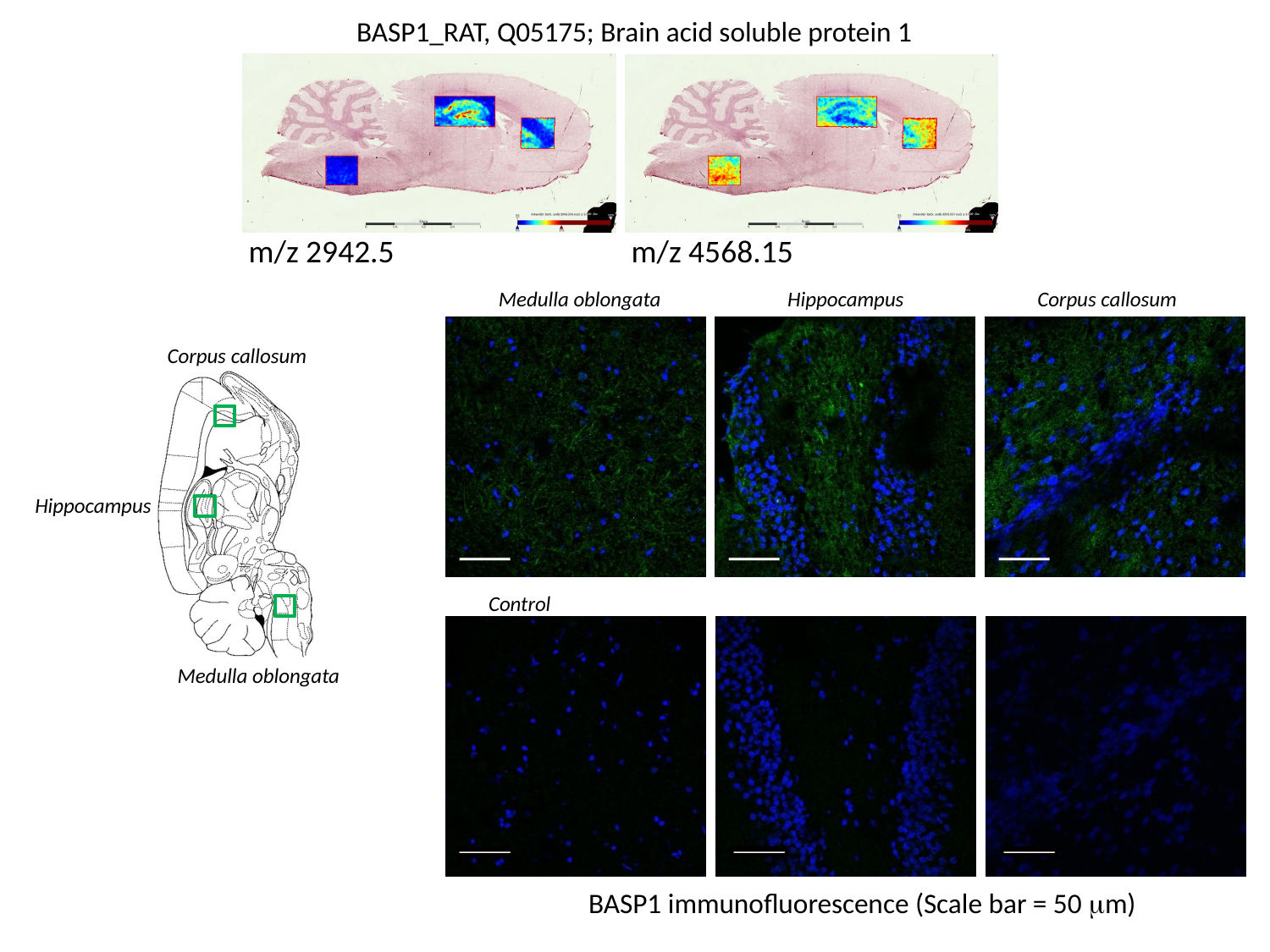

BASP1_RAT, Q05175; Brain acid soluble protein 1
m/z 2942.5
m/z 4568.15
Corpus callosum
Medulla oblongata
Hippocampus
Corpus callosum
Hippocampus
Control
Medulla oblongata
BASP1 immunofluorescence (Scale bar = 50 μm)
